# Distinct subsets of anti-pulmonary autoantibodies correlate with disease severity and survival in severe COVID-19 patients

**DOI:** 10.1007/s11357-023-00887-2

**Published:** 2023-09-01

**Authors:** Emese Tóth, Miklós Fagyas, Béla Nagy, Ivetta Mányiné Siket, Blanka Szőke, Lilla Mártha, Mohamed Mahdi, Gábor Erdősi, Zsófia Pólik, János Kappelmayer, Zoltán Papp, Attila Borbély, Tamás Szabó, József Balla, György Balla, Attila Bácsi, Zoltán Szekanecz, Péter Bai, Attila Tóth

**Affiliations:** 1 Department of Medical Chemistry, Faculty of Medicine, University of Debrecen, 4032 Debrecen, Hungary; 2 Center of Excellence, The Hungarian Academy of Sciences, Budapest, Hungary; 3 Division of Cardiology, Department of Cardiology, Faculty of Medicine, University of Debrecen, Debrecen, Hungary; 4 Division of Clinical Physiology, Department of Cardiology, Faculty of Medicine, University of Debrecen, Debrecen, Hungary; 5 Department of Laboratory Medicine, Faculty of Medicine, University of Debrecen, Debrecen, Hungary; 6 Department of Biochemistry and Molecular Biology, Faculty of Medicine, University of Debrecen, Debrecen, Hungary; 7 ELKH-UD Vascular Biology and Myocardial Pathophysiology Research Group, Hungarian Academy of Sciences, Budapest, Hungary; 8 Research Center for Molecular Medicine, Faculty of Medicine, University of Debrecen, 4032 Debrecen, Hungary; 9 Department of Pediatrics, Faculty of Medicine, University of Debrecen, Debrecen, Hungary; 10 Department of Internal Medicine, Faculty of Medicine, University of Debrecen, Debrecen, Hungary; 11 Department of Immunology, Faculty of Medicine, University of Debrecen, Debrecen, Hungary; 12 Department of Rheumatology, Faculty of Medicine, University of Debrecen, Debrecen, Hungary; 13 MTA-DE Cell Biology and Signaling Research Group ELKH, Debrecen, 4032 Hungary; 14 MTA-DE Lendület Laboratory of Cellular Metabolism, 4032 Debrecen, Hungary

**Keywords:** Lung, Autoantibody, IgM, IgG, Multi-producer, Severe COVID-19, Mortality, Clinical outcome, Post-COVID, SARS-CoV-2

## Abstract

Autoantibodies targeting the lung tissue were identified in severe COVID-19 patients in this retrospective study. Fifty-three percent of 104 patients developed anti-pulmonary antibodies, the majority of which were IgM class, suggesting that they developed upon infection with SARS-CoV-2. Anti-pulmonary antibodies correlated with worse pulmonary function and a higher risk of multiorgan failure that was further aggravated if 3 or more autoantibody clones were simultaneously present (multi-producers). Multi-producer patients were older than the patients with less or no autoantibodies. One of the identified autoantibodies (targeting a pulmonary protein of ~ 50 kDa) associated with worse clinical outcomes, including mortality. In summary, severe COVID-19 is associated with the development of lung-specific autoantibodies, which may worsen the clinical outcome. Tissue proteome-wide tests, such as the ones applied here, can be used to detect autoimmunity in the post-COVID state to identify the cause of symptoms and to reveal a new target for treatment.

## Introduction

The extreme immune system activation occurring in severe SARS-CoV-2 infection may lead to long-lasting nonspecific symptoms, referred to as long or post-COVID syndrome [1, 2]. Some of these symptoms are the direct result of unrepairable organ damage during the acute phase of coronavirus disease 2019 (COVID-19), while others seem to be independent of that. These latter cases may be explained by the initiation of autoimmune pathologies during the acute phase of COVID-19. Indeed, SARS-CoV-2 infection initiates the development of autoantibodies [3–9], including those associated with systemic autoimmune rheumatic diseases [10], as well as interferons [11].

Some of these autoantibodies were implicated in COVID-19 mortality by suppressing immune responses [12]. Nevertheless, it is likely that autoantibodies in COVID-19 affecting the clinical outcome may not be limited to the elements of the immune system. Indeed, testing the whole extracellular and secreted proteome, a wide range of autoantibodies were found [13]⁠, suggesting an untargeted, general humoral response. Development of anti-cardiac autoantibodies correlated with the (nonspecific) cardiac damage in severe acute COVID-19, suggesting that organ damage may play an important role in the initiation of autoantibodies by providing potential intracellular antigens in large quantities [14].

SARS-CoV-2, the virus that causes COVID-19, primarily targets the upper and lower respiratory tract. The primary target of SARS-CoV-2 is the angiotensin-converting enzyme 2 (ACE2) receptor, which is present on many types of cells in the body, including cells in the lung [15]. Virus invasion and replication within cells may lead to direct organ damage and hyper-inflammation in the lungs.

Here, we tested the appearance of anti-pulmonary autoantibodies in the hyperinflammatory lungs of severe COVID-19 patients. We used a tissue-wide proteome bait (i.e., whole human lung homogenate) to detect all potential autoantibodies, and tested both IgG and immunoglobulin (Ig)M classes. We found that more than half of the severe COVID-19 patients developed autoantibodies against various pulmonary proteins. Most of these autoantibodies belonged to the IgM class, suggesting an acute COVID-19-related development. A number of autoantibody clones had been associated with organ damage and older age, while some other clones (recognizing a distinct pulmonary protein) might be associated with mortality.

## Results

### The presence of lung-specific autoreactive antibodies correlates with worse clinical outcome

**Fig. 1:**
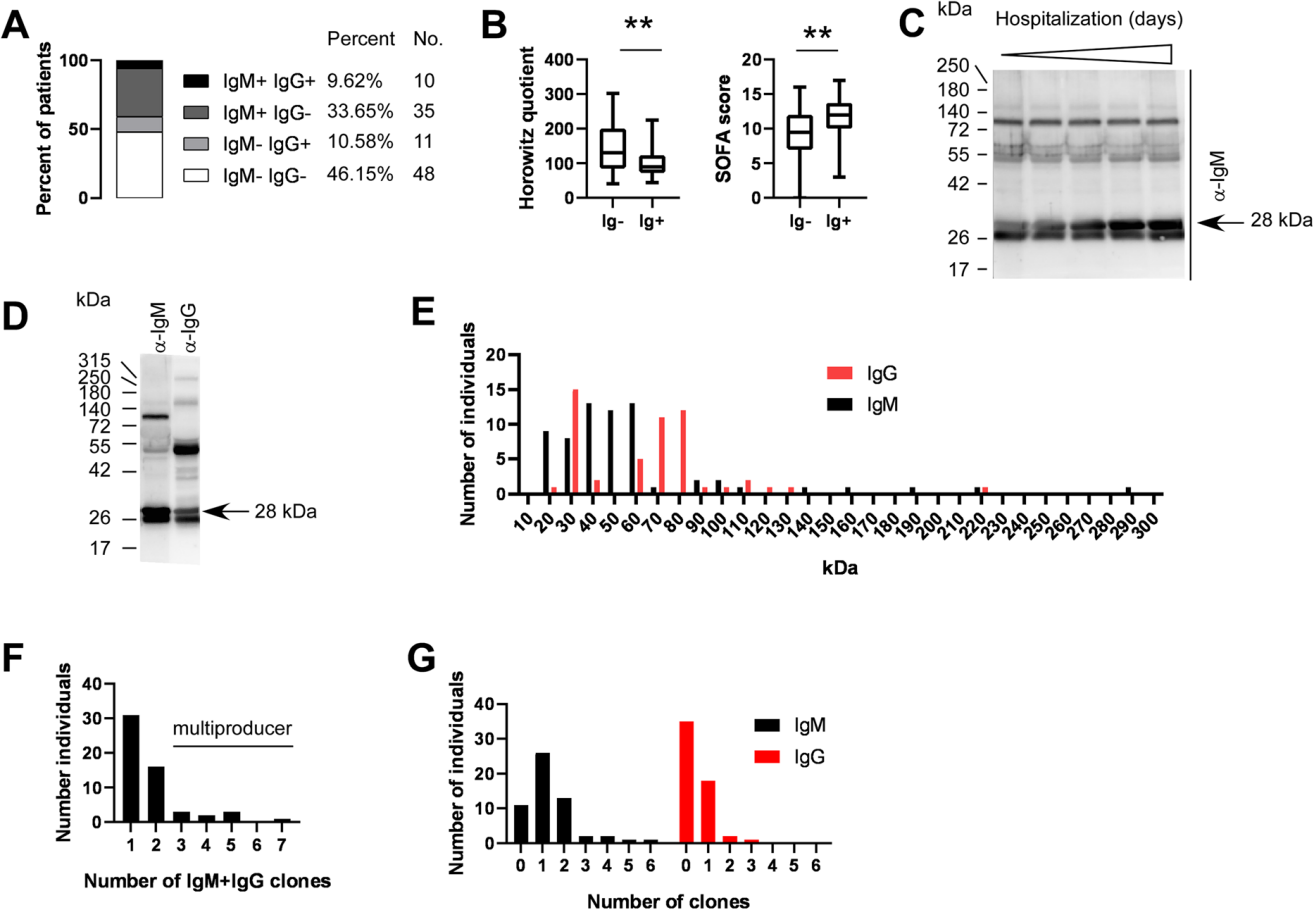
Autoantibody production in COVID-19 patients worsen the clinical status of the disease. **A** The proportions of the IgM and/or IgG positive patients are provided among COPD patients (denoted as controls) and COVID-19 patients. **B** The Horowitz quotient and the SOFA score of the immunoglobulin-negative (Ig −) and immunoglobulin-positive (Ig +) patients. Data cleaned of outliers are presented on box-whiskers diagrams. Asterisks (**) represent statistically significant differences among the groups at *p* < 0.01 using Student’s *t*-test. **C** A representative image of the time course of autoantibody production during hospitalization of a patient. **D** Sign of IgM to IgG transition in a patient. **E** Histogram of the distribution of the apparent molecular mass of the targets of the immunoglobulins. Bin size = 10 kDa. **F** Histogram of the number of all autoantibody clones in a patient. **G** Histogram of the number of IgM and IgG autoantibody clones in a patient

We identified lung-specific autoantibodies in severe COVID-19 patients. Half of the patients (53.85%) developed IgM or IgG antibodies (Fig. 1A and Table 1). The majority of these autoantibodies were of IgM isotype (43.27%) suggesting that these B cell clones developed during the course of the current COVID-19 infection (Fig. 1A and Table 1). The proportions of Ig + patients among COVID-19 patients (53.85%) is drastically larger than among COPD patients (28.57%) who were taken as controls (Fig. 1A and Table 1).

**Table 1:** Percent composition of the immunoglobulin producer patients among COVID-19 patients and non-COVID-19, non-autoimmune lung patients. Number of patients and the percent as of the total of the group (in brackets) is presented

	IgM − IgG-	IgM − IgG +	IgM + IgG-	IgM + IgG +
COVID-19 patients	48 (46.15%)	11 (10.58%)	35 (33.65%)	10 (9.62%)
COPD patients	15 (71.43%)	2 (9.52%)	1 (4.76%)	3 (14.29%)

Next, we compared the Horowitz index, a readout of lung function and SOFA score, a value indicating multiorgan failure, of the immunoglobulin-negative and immunoglobulin-positive patients without discriminating between IgM and IgG isotypes. Immunoglobulin-positive patients had lower Horowitz quotient than immunoglobulin-negative patients, indicating more severe lung damage (Fig. 1B). Furthermore, SOFA score of the immunoglobulin-positive patients was higher than the immunoglobulin-negative ones, signifying a worse overall condition of patients (Fig. 1B). Apparently, the presence of lung-specific autoantibodies coincides with a worse clinical picture of the patients. For certain patients, we observed a time-dependent increase in the titer of an autoantibody (Fig. 1C); however, in other cases, the intensity of the autoantibodies did not change during the course of hospitalization. These antibodies are likely natural antibodies generated by B1 cells [16]. Furthermore, in the case of one patient, we observed the transition of an IgM clone to an IgG clone (Fig. 1D).

The protein targets of the IgM antibodies were in the range of 20–60 kDa, being somewhat different than that of the IgG (30–80 kDa range) (Fig. 1E). Nevertheless, there were targets with higher molecular weight in a small number of patients.

Most patients had 1 or 2 autoreactive clones, while a minority of the patients had 3 or more autoreactive clones. The highest number of clones observed was 7 (Fig. 1F). For patients with 3 or more autoreactive clones, we used the collective term “multi-producer.” When comparing the number of IgM and the IgG clones in the case of one patient, we observed that the maximal number of IgM clones was higher than the maximal number of IgG clones (6 vs. 3) (Fig. 1G).

**Fig. 2:**
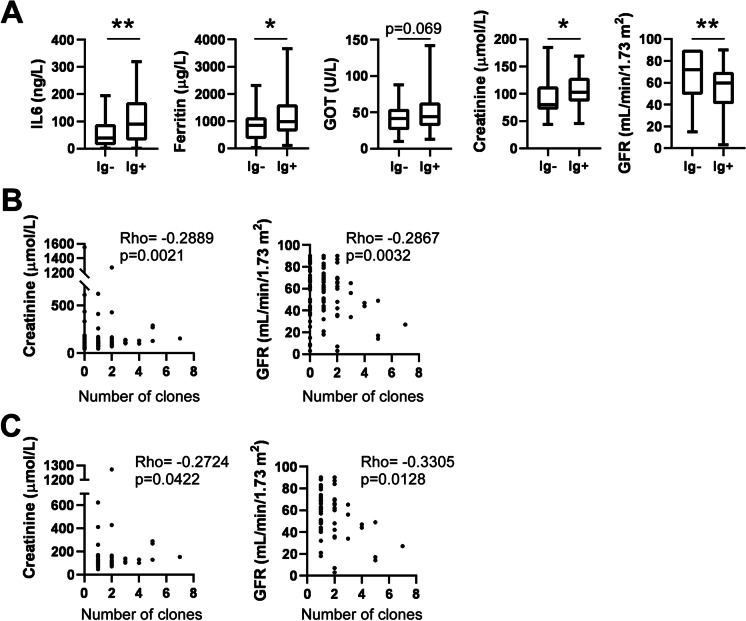
Autoantibody production positively correlate with markers of tissue and organ damage. **A** Serum chemistry data of the immunoglobulin-negative (Ig −) and immunoglobulin-positive (Ig +) patients is presented. Data cleaned of outliers are presented on box-whiskers diagrams. Asterisks (* and **) represent statistically significant differences among the groups at *p* < 0.05 and *p* < 0.01, respectively, using Student’s *t*-test. **B** and **C** Correlation between the number of all immunoglobulin clones in a patient and serum creatinine or glomerular filtration rate (GFR) was assessed using Spearman correlation on all patients (**B**) or on only patients producing autoantibodies (note, missing values at “0” clones, **C**)

We compared serum chemistry parameters of the immunoglobulin-positive and -negative patients. Importantly, markers of higher immune reaction, such as serum interlekin-6 (IL-6) levels, serum ferritin, serum glutamic oxaloacetic transaminase (GOT) levels, and kidney function markers (serum creatinine, glomerular filtration rate (GFR)), were higher in the immunoglobulin-positive patients as compared to immunoglobulin-negative patients (Fig. 2A). Serum creatinine levels and GFR values of the patients correlated with the number of autoreactive clones among all patients (i.e., immunoglobulin positive + negative patients) (Fig. 2B), as well as among the immunoglobulin-positive cases (Fig. 2C).

### Patients with multiple anti-lung autoreactive clones have adverse clinical outcome

**Fig. 3:**
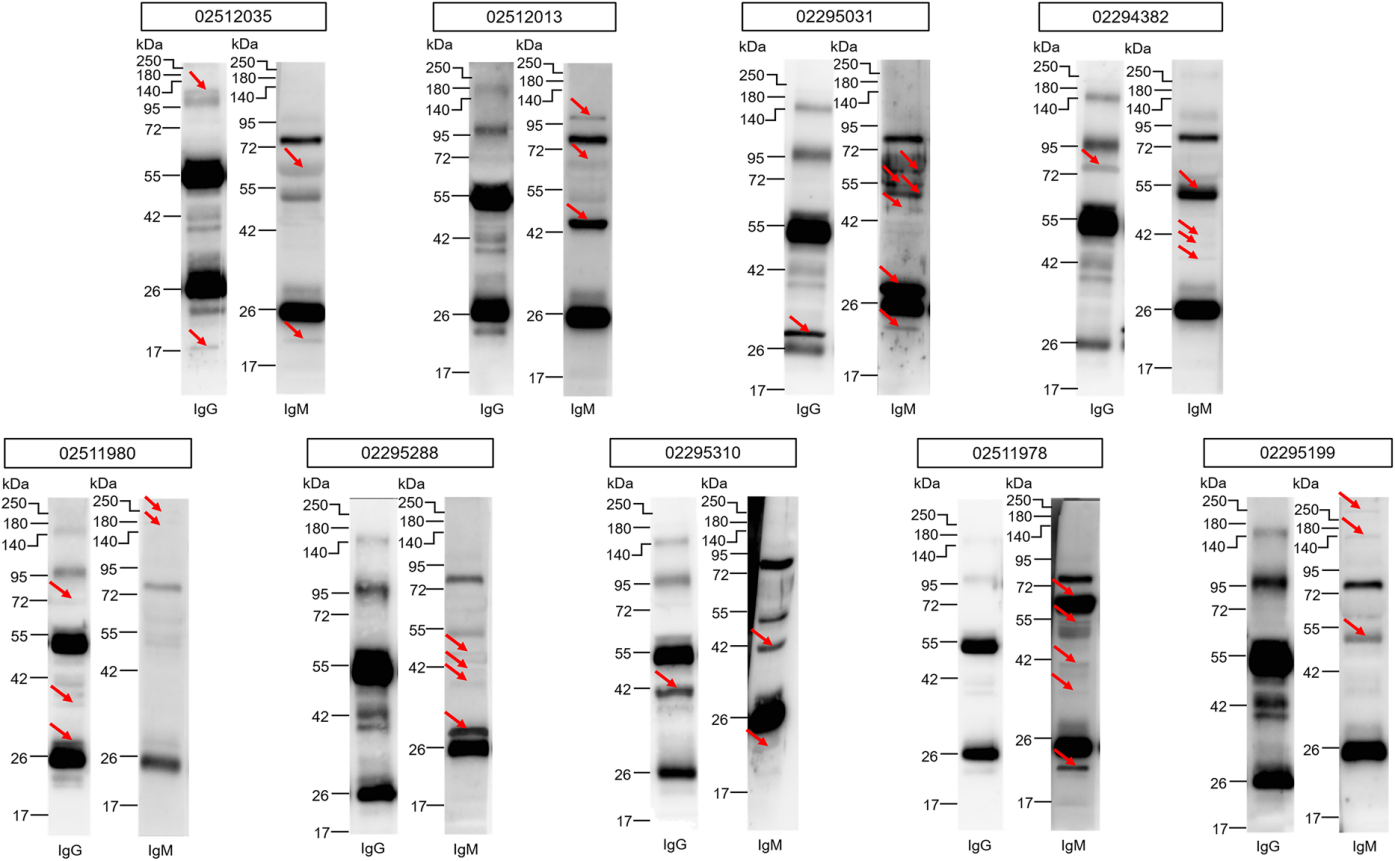
The representation of the IgM and IgG autoantibodies of the multi-producer patients identified in this study. Lung-specific IgM and IgG autoantibodies were detected as described in the Materials and Methods. All multi-producer patients are presented in the figure. Red circles indicate the location of the anti-lung IgM or IgG bands

**Fig. 4:**
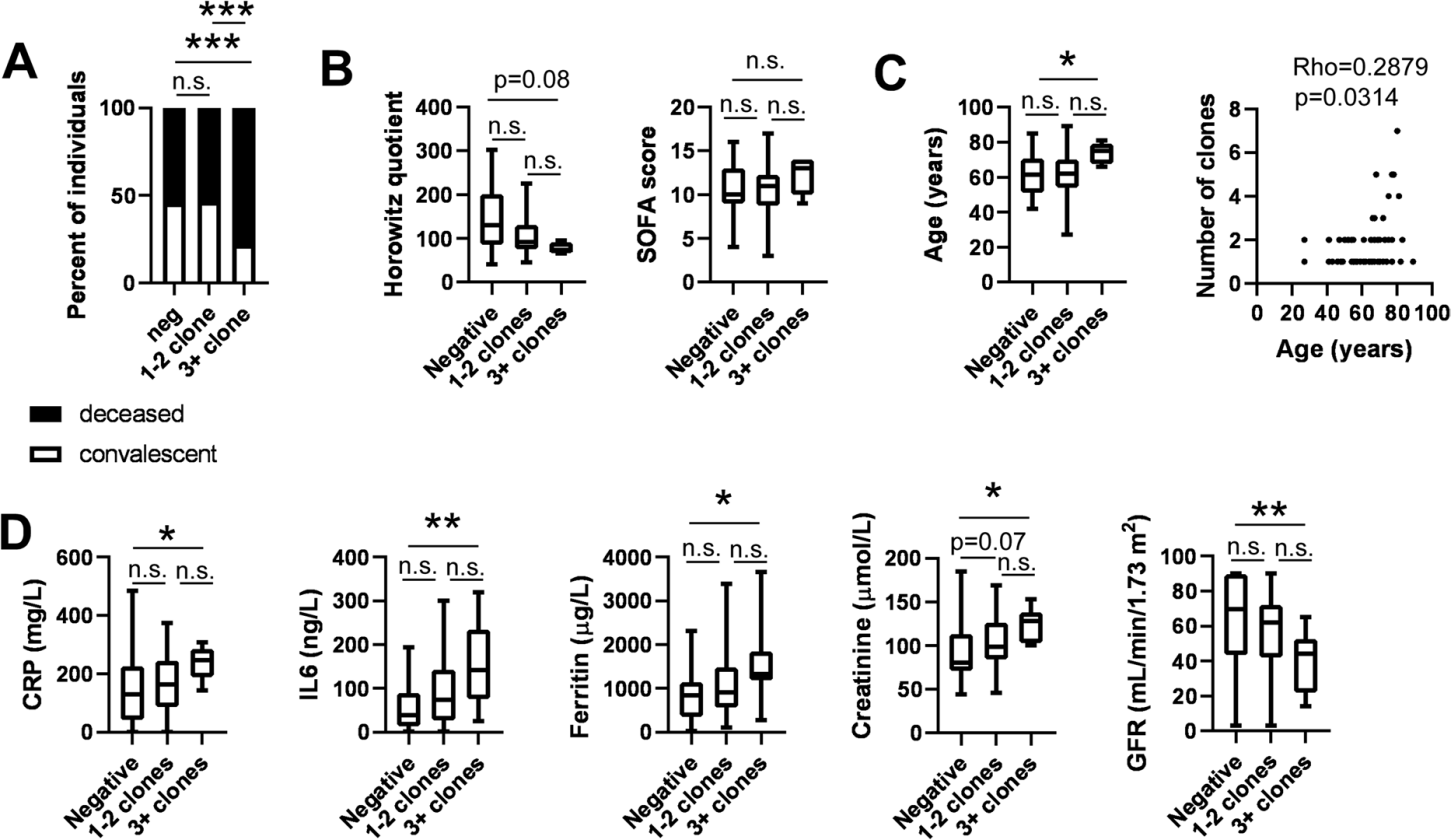
Multi-producer patients develop more severe COVID-19 disease. Patients enrolled in the study were stratified as (i) autoantibody negative, (ii) 1–2 autoantibody clones, and (iii) 3 or more autoantibody clones (multi-producers) using human lung tissue proteome as bait. Among these 3 groups, **A** the pattern of disease outcome, **B** the Horowitz quotient and the SOFA score, **C** patient age, and **D** serum chemistry readouts were compared. On panel **A**, chi-square test was applied, and *p* values were normalized for multiple comparisons. On panels **B** and **D**, data was cleaned of outliers and normality was checked. Subsequently, one-way ANOVA test was used followed by a post hoc test as a function of the normality of the values. Information on normality and the post hoc test can be found in the shared primary data files. Data are presented on box-whiskers diagrams. Asterisks represent statistically significant differences among the groups at *p* < 0.05 (*), *p* < 0.01 (**), and *p* < 0.001 (***). On panel **C**, the correlation between the number of all immunoglobulin clones and patient age was assessed using Spearman correlation. Abbreviation: n.s. not significant

We stratified patients as a function of the number of lung-specific autoreactive clones yielding a group with no autoreactive clones, 1–2 autoreactive clones, and 3 or more autoreactive clones (multi-producer patients) (Fig. 3). We have not identified multi-producers in the COPD (control) group. While the proportions of the deceased versus convalescent patients were not different between the immunoglobulin-negative patients and the patients with 1–2 clones, the ratio of the deceased patients was significantly higher among the multi-producer patients than among the immunoglobulin-negative and the 1–2 antibody groups (Fig. 4A). In good agreement with that, the Horowitz quotient of the multi-producer patients was lower than the immunoglobulin-negative and the group expressing 1–2 antibodies (Fig. 4B). Similarly, the SOFA score of the multi-producer patients was higher than the immunoglobulin-negative and the 1–2 antibody group (Fig. 4B).

Importantly, the multi-producer patients were older than those who produced 1–2 autoantibodies and the non-producers (Fig. 4C). Age correlated with the number of autoantibody clones present (Fig. 4C). Multi-producer patients had worse inflammatory conditions, marked by higher serum C-reactive protein (CRP) and IL-6 levels, as compared to patients with maximum 2 autoantibody clones (Fig. 4D). Furthermore, serum ferritin levels were higher in multi-producer patients as compared to patients with up to 2 autoantibody clones (Fig. 4D). Finally, serum creatinine levels were also higher, while GFR was lower in multi-producer patients as compared to patients with up to 2 autoantibody clones, evidencing correlation with impaired kidney function (Fig. 4D).

### Only a subset of autoantibodies affects disease outcome in severe COVID-19 patients

We assessed the correlation between the molecular targets of the autoantibodies and the clinical outcome of the disease. To that end, we stratified patients as a function of the apparent molecular mass of the protein targets of the autoantibodies, and we treated IgM and IgG antibodies as separate entities even if the apparent molecular weight of the targets were similar, with the exception of high molecular weight targets due to the little number of targets in that range. For each set of autoantibodies, we assessed the proportions of the convalescent and deceased individuals among (1) autoantibody-negative patients, (2) autoantibody-positive patients, lacking antibody against the indicated target, (3) patients exclusively producing autoantibody or autoantibodies against the indicated target, and (4) patients with multiple autoantibodies including one(s) recognizing the indicated target.

**Fig. 5:**
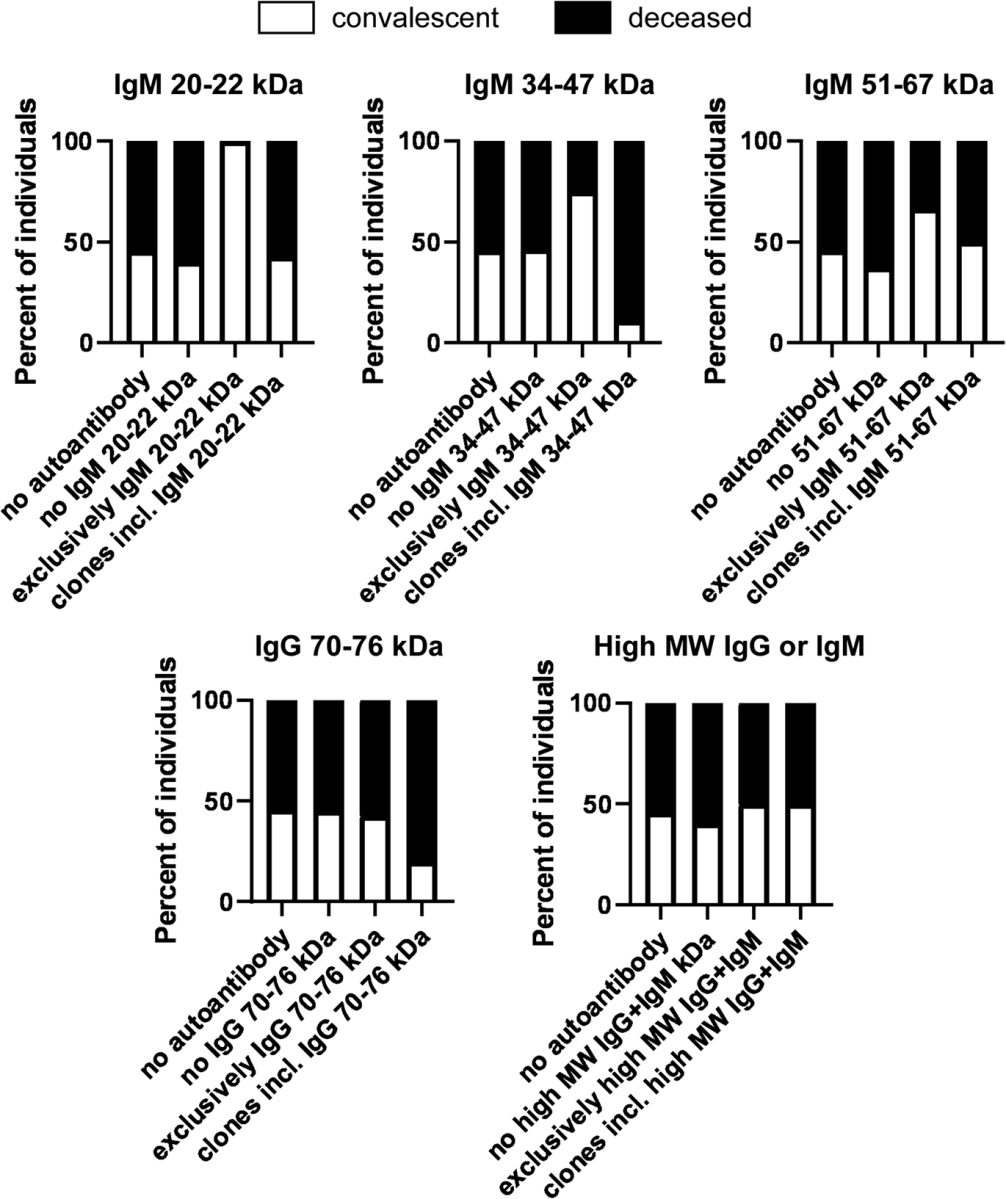
Certain molecular targets of the autoantibodies do not associate with disease outcome. Patients enrolled into the study were stratified as indicated on the panels. To assess differences in the distribution of the convalescent and deceased patients, chi-square test was applied, and *p* values were normalized for multiple comparisons

**Fig. 6:**
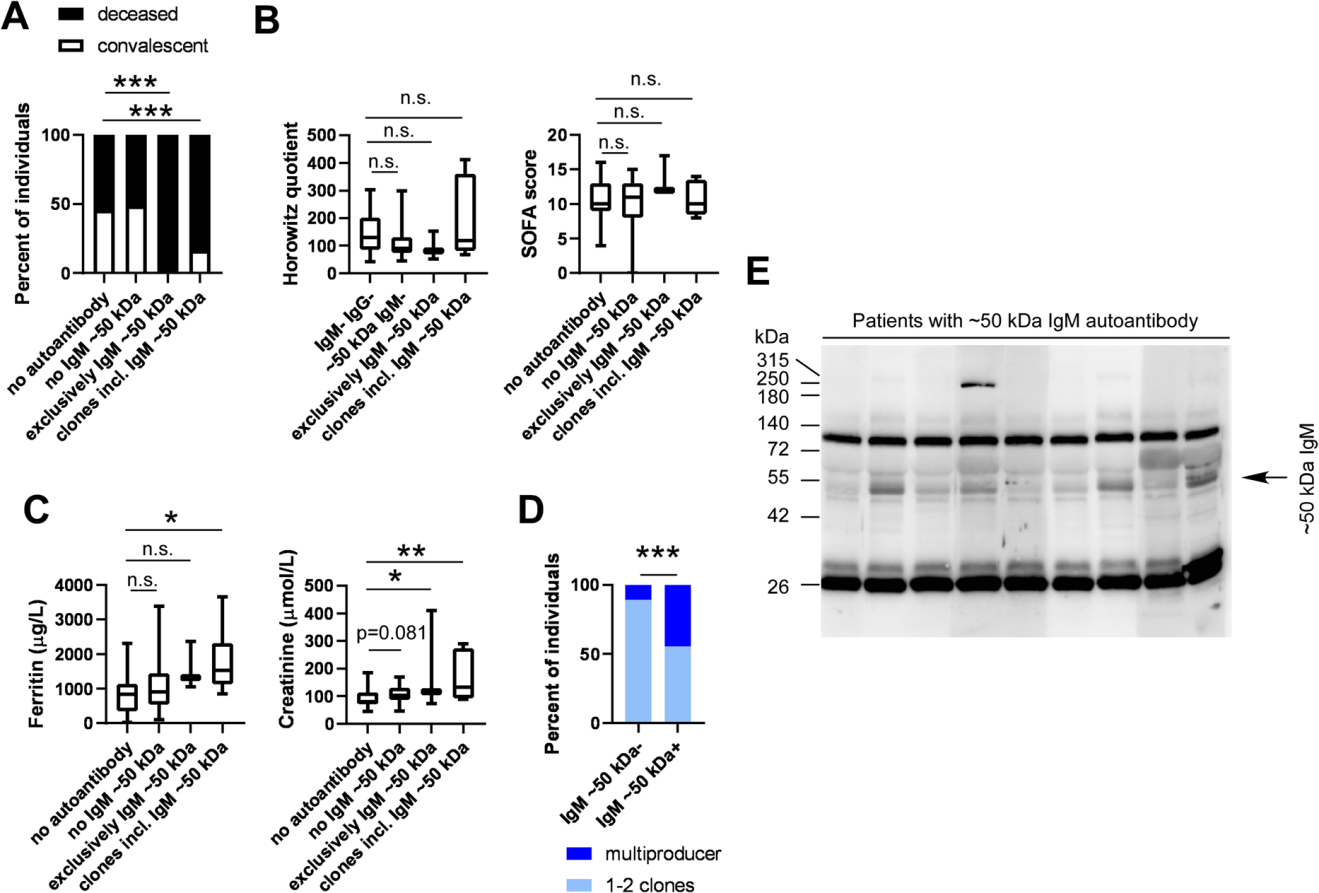
Autoantibody-recognizing targets with a molecular mass of ~ 50 kDa associate with poor survival. Patients enrolled in the study were stratified as indicated on the panels. **A** The proportions of the deceased and convalescent patients were plotted, and statistical evaluation was performed on the absolute number of patients. For the same patient groups, **B** the Horowitz quotient, the SOFA score, and **C** serum chemistry readouts were compared. **D** The proportions of the multi-producer and non-multi-producer patients were determined. **E** A blot image of the autoantibodies recognizing the ~ 50 kDa target in all patients positive for this autoantibody. On panels **A** and **D**, chi-square test was applied, and *p* values were normalized for multiple comparisons. On panels **B** and **C**, data was cleaned of outliers, and normality was checked. Subsequently, one-way ANOVA test was used followed by a post hoc test as a function of the normality of the values. Information on normality and the post hoc test can be found in the shared primary data files. Data are presented on box-whiskers diagrams. Asterisks represent statistically significant differences among the groups at *p* < 0.05 (*), *p* < 0.01 (**), and *p* < 0.001 (***). Abbreviation: n.s. not significant

In most cases (IgM 20–22 kDa, IgM, 34–47 kDa, IgM 51–67 kDa, IgG 71–76 kDa, high molecular weight IgM + IgG), there was no difference in disease outcome that can be specifically attributed to the presence of an autoantibody or autoantibodies (Fig. 5 and Table 2). Nevertheless, we have identified three targets that specifically associated with worse clinical outcome. The worst proportions of the deceased vs. convalescent patients were observed in the case of IgM autoantibody or autoantibodies in the range of ~ 50 kDa. We found only deceased patients among those who produced only IgM autoantibodies with a ~ 50 kDa target. Similarly, among the patients with multiple autoantibody clones that included an IgM autoantibody with ~ 50 kDa IgM target, we observed higher proportions of deceased patients (Fig. 6A). Patients producing the ~ 50 kDa IgM autoantibody or autoantibodies were characterized by lower Horowitz quotient and higher SOFA score (Fig. 6B), both suggesting an overall severe course of the disease. In good agreement with that, serum ferritin and creatinine levels were higher in patients expressing the autoantibody in question (Fig. 6C). Furthermore, we preferentially found multi-producers among patients expressing the autoantibody with a ~ 50 kDa target (Fig. 6D). When patient serum samples containing the ~ 50 kDa IgM antibody were probed using the same blot, there was little to no variability among the molecular weight of the target of the autoantibody (Fig. 6E). More male patients were found among those expressing the IgM autoantibody with ~ 50 kDa target (77.78% of males (7 males vs. 2 females) vs. 64.42% of males (67 males vs. 37 females) among the study population or 67.86% of males (38 males vs. 18 females) among the immunoglobulin-positive patients). None of the Ig + patients among the COPD patients (controls) produced immunoglobulins recognizing targets with an apparent molecular weight of ~ 50 kDa.

**Table 2:** The number of patients as a function of the molecular mass of the target of the autoantibodies in the COVID-19 patient cohort. Those molecular mass ranges are marked in bold that affect patient survival

Target	Patients negative for the target		Patients with only the Ig against the target		Patients with Ig against the target among other Igs	
	Conv	Deceased	Conv	Deceased	Conv	Deceased
IgM 20–22 kDa	19	28	2	0	3	4
IgM ~ 28 kDa	**21**	**27**	**1**	**3**	**2**	**2**
IgM, 34–47 kDa	20	23	3	1	1	8
IgM ~ 50 kDa	**44**	**21**	**0**	**3**	**1**	**4**
IgM 51–67 kDa	15	25	4	2	5	5
IgG ~ 28 kDa	**22**	**26**	**1**	**2**	**1**	**4**
IgG 71–76 kDa	20	24	3	4	1	4
High MW IgM + IgG (> 80 kDa)	17	25	2	2	5	5
Number of Ig − patients	22	26

**Fig. 7:**
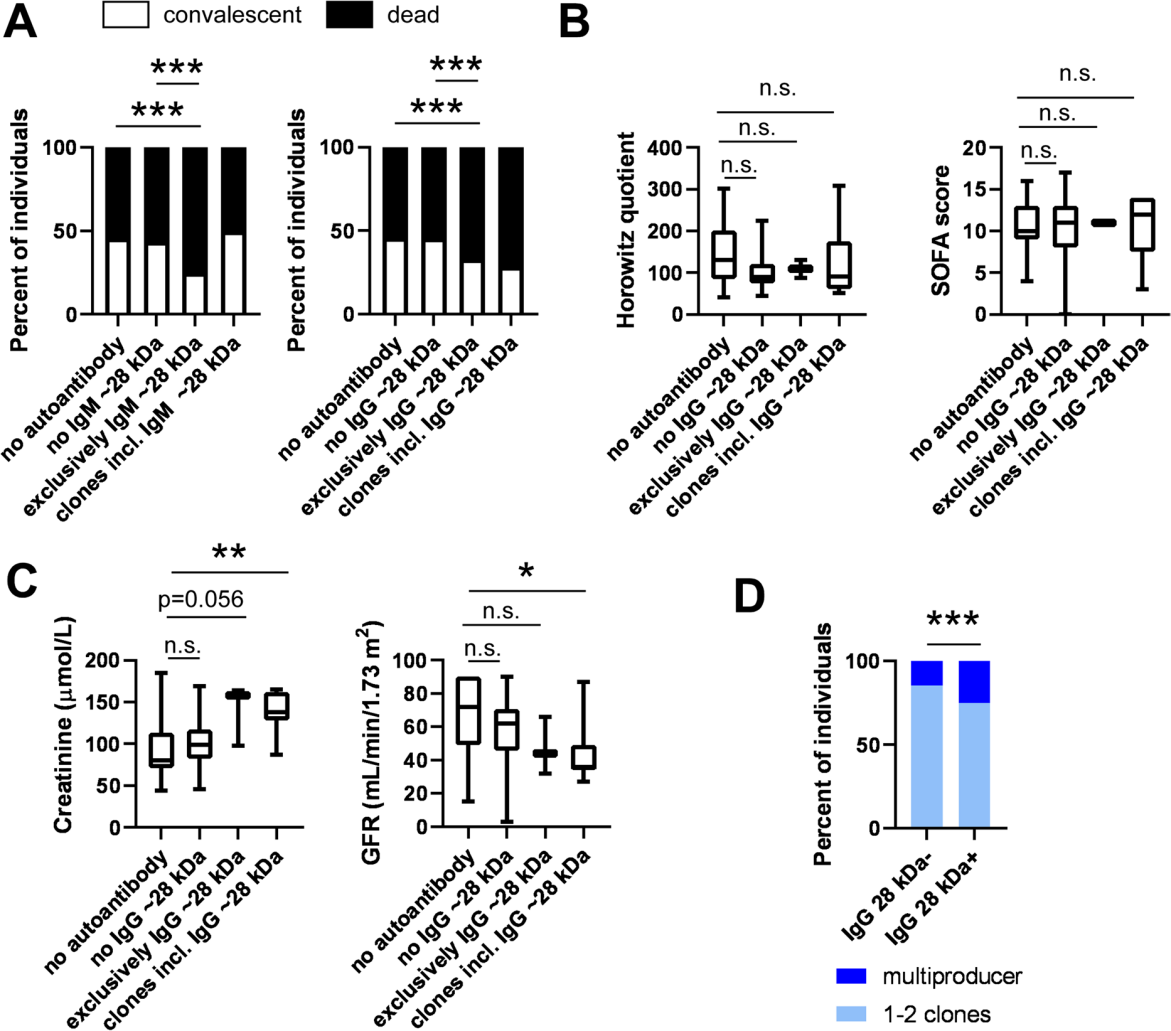
Autoantibody-recognizing targets with a molecular mass of ~ 28 kDa associate with poor survival. Patients enrolled into the study were stratified as indicated on the panels. **A** The proportions of the deceased and convalescent patients were plotted and statistical evaluation was performed on the absolute number of patients. For the same patient groups, **B** the Horowitz quotient, the SOFA score, and **C** serum chemistry readouts were compared. **D** The proportions of the multi-producer and non-multi-producer patients were determined. On panels **A** and **D**, chi-square test was applied, and *p* values were normalized for multiple comparisons. On panels **B** and **C**, data was cleaned of outliers and normality was checked. Subsequently, one-way ANOVA test was used followed by a post hoc test as a function of the normality of the values. Information on normality and the post hoc test can be found in the shared primary data files. Data are presented on box-whiskers diagrams. *, **, and *** represent statistically significant differences among the groups at *p* < 0.05, *p* < 0.01, and *p* < 0.001 using between the groups indicated. Abbreviation: n.s. not significant

Another set of autoantibodies that associated with poor survival recognized various targets in the molecular weight range of ~ 28 kDa. The production of both IgM and IgG autoantibodies were associated with worse clinical outcome of the disease (Fig. 7A). It is important to note that in the case of one patient, we observed signs of an IgM to IgG transition of a clone with a target of ~ 28 kDa (Fig. 1D). Although the changes did not reach statistical significance, patients producing IgG autoantibodies had lower Horowitz quotient and similar SOFA score as compared to autoantibody-negative COVID-19 patients (Fig. 7B). Furthermore, patients producing the ~ 28 kDa IgG autoantibody had higher serum creatinine levels and lower GFR values as compared to the patients not producing the autoantibodies (Fig. 7C). In addition, we preferentially found multi-producers among patients expressing the IgG autoantibody with ~ 28 kDa target (Fig. 7D). More male patients were found among those expressing the IgG autoantibody with ~ 28 kDa target (87.50% of males (7 males vs. 1 female) vs. 64.42% of males (67 males vs. 37 females) among the study population or 67.86% of males (38 males vs. 18 females) among the immunoglobulin-positive patients). One COPD (control) patient was identified with an IgM clone recognizing a target protein of ~ 28 kDa.

## Discussion

Viruses have long been shown to transiently perturb the immune system, leading to its dysregulation and the generation of autoantibodies [17, 18]. Molecular mimicry and cross-reactivity between viral and tissue antigens, direct cytopathic effects of viral replication and apoptosis, and dysregulation of B-cells have all been proposed as pathomechanisms for their development [19, 20]. To mention few examples, infection with Epstein-Barr virus has been associated with higher frequency of autoantibodies reacting with cardiolipin and cytoskeletal proteins. Autoantibodies directed against liver and kidney tissues were also described in the context of infection with viral hepatitis [21]. On the other hand, many patients infected with the human immunodeficiency virus (HIV) were found to have a wide range of autoantibodies, reacting against platelets, and erythrocytes, smooth muscle, in addition to antinuclear antibodies, rheuma factor, and anti-phospholipid antibodies [22]. More recently, antinuclear/extractable-nuclear antibodies and autoantibodies reacting with type I IFNs, Anti-Platelet Factor 4, were associated with COVID-19 [3–11], although causation or correlation has not yet been established with the infection.

Here, we tested the appearance of lung-specific autoantibodies in severe COVID-19 patients during their hospital care. More than half of these patients featured one or more clones of autoantibodies, recognizing human pulmonary proteins. Mortality increased among patients with at least 3 autoantibody clones recognizing different pulmonary proteins, similar to previous observations linking autoimmunity to disease severity [23]. In addition, multi-producer patients were elder than the mean age of the patients enrolled which is in good correlation with previously published data [4]. Of note, the study represents correlations between age and the number of autoantibody clones that cannot be considered an established causative relationship, nevertheless, other studies have already established that autoimmunity is more likely among the elderly (e.g., [24]).

These results show that anti-pulmonary autoantibodies are frequently developing in acute COVID-19 patients, over 50% of the study population. It appears that excessive tissue damage and hyper-inflammation in severe acute COVID-19 promote autoantibody production. It is not clear whether these autoantibodies are only biomarkers of general organ damage in severe acute COVID-19, or whether they also contribute to the deterioration of physiological functions and worsening of the patient’s condition.

Studies have already identified multiple targets of autoantibodies as anti-cardiac autoantibodies [14], autoantibodies to antigens related to systemic autoimmune rheumatic conditions [10], interferons [11], and other targets [3–7]. In our study, we also observed a multitude of autoantibodies against the lung tissue and importantly, the distribution of autoantigens was not random, a definite accumulation of some specific targets was seen in the case of pulmonary autoantibodies. In other words, some of the pulmonary proteins proved to be better immunogens. This observation is in contrast to our previous study on cardiac autoantibodies as, apparently, there are no apparent preferred targets in the case of the cardiac tissue [14].

Importantly, the presence of anti-lung autoantibodies showed correlation with mortality and worse clinical picture of the disease, in contrast to anti-cardiac autoantibodies that did not associate with mortality [14]. Moreover, we identified a specific ~ 50 kDa antigen in the pulmonary tissue that was highly associated with mortality. This suggests that there may be a molecular mimicry between the pulmonary protein and the SARS-CoV-2 (facilitating autoantibody production), or we have prevalence of patients with this specifically targeted protein. This question cannot be decided based on the available data, but it is intriguing that individuals with the ~ 50 kDa antigen may have more severe symptoms, therefore, presenting at the emergency care unit, from where most of our patients were recruited.

In a previous publication [14], we performed a similar analysis than that in the present manuscript on heart tissue homogenates. We found a similar level of autoimmunity (more, than half of the patients harbored anti-cardiac autoantibodies). Nonetheless, we noted three important differences: first, in most cases, the anti-lung and anti-cardiac antibodies recognized different molecular targets (different sizes of autoantigens); second, the presence of anti-cardiac autoantibodies did not correlate with mortality; and third, anti-cardiac autoantibodies did not recognize a cluster of similarly sized autoantigens. All these differences suggest that the anti-pulmonary autoantibodies are targeting specifically the lungs.

There are limitations to this study. It represents patients infected by the delta strain, and it is most likely that different strains of SARS-CoV-2 induce different immune responses. This is a retrospective study performed in a population with severe COVID-19; of note, about half of the involved patients died. We do not have information on the titer of the SARS-CoV-2 at the time of blood sampling, making us unable to correlate the autoimmunity with the severity of infection. This did not allow identifying certain variables, such as the time of infection and the time of hospitalization relative to that. The lack of sufficient human biological samples precluded the identification of the antigens. Another limitation of the study is that with the available methodology, we cannot determine the affinity of the autoantibodies, as the signal on blots does not necessarily correlate with binding affinity but is affected by the quantity of the autoantibodies in serum and the quantity of the antigens in the lung tissue homogenate used as bait.

Independently of their role in mortality, novel autoantibodies generated during COVID-19 may re-activate after the acute phase of the disease and can cause symptoms related to the targeted organ, contributing to long (or post-) COVID syndrome. The occurrence of anti-pulmonary autoantibodies (54% among the severe COVID-19 patients) correlated well with the observed occurrence of long-COVID syndrome in severe (up to 83%) [25] and in mild (up to 35%) COVID-19 patients [26] during the same phase (delta strain) of COVID-19 pandemic. It appears that autoimmunity may be an important contributor to long COVID-19, and may require an adequate, targeted therapeutic approach to treatment.

## Significance

In this study, we showed that half of the patients with severe COVID-19 produce anti-pulmonary autoantibodies, the majority of which were IgM class autoantibodies. The presence of autoantibodies correlated with worse pulmonary function that was aggravated in a subset of patients characterized by the simultaneous presence of 3 or more autoantibody clones. These patients were than the patients with less or no autoantibodies. Importantly, we identified two autoantibodies that associated with worse clinical outcomes, including mortality. These autoantibodies may promote autoimmune reactions, which can complicate post-COVID recuperation, contributing to post-acute sequelae of COVID-19 (long COVID).

## STAR methods

### Key resources table

**Table:** 

Reagent or resource	Source	Identifier
Nitrocellulose membrane 0,45um	Bio-Rad Laboratories	CAT: 1,620,115 LOT: A17567266
Anti-Human IgM (Fc5μ)-Peroxidase antibody produced in rabbit	Merck	CAT: SAB3701404 LOT: RI34003
Peroxidase- conjugated Affini Pure Donkey Anti- Human IgG (H + L)	Jackson Immuno Research Laboratories	CAT: 709,035,149 LOT: 70,065
Western Lightning Plus- ECL	Perkin Elmer	CAT: NEL105001EA LOT: 203–12431
ProSieve QuadColor protein marker	Lonza	CAT: 001938837
ImageLab software	Bio-Rad Laboratories	v 6.1.0
GraphPad Prism	GraphPad	v 8.0.1

### Contacts for reagent and resource sharing

All requests for reagents and resources should be directed to the lead contacts, Attila Tóth (atitoth@med.unideb.hu).

### Patient recruitment

Patient recruitment, demographic, and laboratory analyses were published earlier [14]. Serum samples of patients with COPD were collected at the Institute of Laboratory Medicine at the University of Debrecen. The characteristics of the patient cohorts are in Table 3.

**Table 3:** Characteristics of patient cohorts

	Patients with COVID-19	COPD patients
Number of patients	104	21
Age (years) (median, IQR)	65 (52.25–72)	61 (53–72)
Male/female, *n*	67/37	11/10
IL-6 (ng/L) (median, IQR)	82 (26–185)	N/A
CRP (mg/L) (median, IQR)	157 (62–240)	2.3 (0.9–5.0)
Ferritin (μg/L) (median, IQR)	952 (557–1603)	86 (42.9–260.5)
GFR-EPI (mL/min/1.73m^2^) (median, IQR)	63 (42–83)	86 (70–90)
Hemoglobin (g/L) (median, IQR)	134 (120–147)	145 (134–152)
White blood cell count (G/L) (median, IQR)	8.91 (6.663–11.80)	7.4 (5.9–8.6)
Diabetes mellitus, *n*, %	36, 35	5, 24
Hypertension, *n*, %	78, 75	11, 52
COPD, *n*, %	14, 13	21, 100
Atrial fibrillation, *n*, %	24, 23	4, 19
Renal insufficiency, *n*, %	23, 22	3, 14
Hypothyreosis, *n*, %	6, 6	1, 5

### Ethical approval

Ethical approvals were issued by the Scientific and Research Ethics Committee of the University of Debrecen and the Ministry of Human Capacities. Recruiting COVID-19 patients was approved under the registration number 32568-32020EÜIG⁠. The human lung samples used for the identification of the autoantibodies were collected and banked under the cover of an ethical permit issued by the Hungarian Ministry of Health UDCC RECIEC 4375–2015.

### Detection of anti‑lung autoantibodies

Autoantibody detection was described earlier [14].

### Determination of the molecular weight of pulmonary autoantigens

For the molecular weight analysis of pulmonary autoantigens, ProSieve QuadColor protein marker (Lonza, Basel, Switzerland, Cat. No. 001938837) was applied. Western blot images were processed using ImageLab software v 6.1.0. (Bio-Rad Hercules, CA, USA). First, lanes and bands were detected, a lane profile was created, representing a line-scan of optical densities. After the background signal has been subtracted, area of the lanes was calculated.

Next, an individual calibration curve was created for each membrane by plotting the molecular weights of standard protein bands (in kDa) against the relative mobility of the bands from the top of the membrane. Data was fit by semi-log regression method. Inaccuracies of SDS gel running were corrected by the adjust frame tool of the ImageLab software. The accurate molecular weights of the autoantigens were determined using the standard curve created for the same membrane using the built-in Molecular Weight Analysis Tool of the software.

### Statistical analysis

Statistical analysis was performed using 8.0.1 version of GraphPad Prism. If necessary, values were cleaned of outliers using the ROUT method. Values were tested for normal distribution using the Shapiro–Wilk normality test. When necessary, values were log normalized. For comparing two groups, *t*-test was used. For comparing multiple groups, ANOVA test was used following a post hoc test as indicated in the figure legends. Chi-square test was performed using Excel; *p* values were corrected for multiple comparisons. To assess correlations, the Spearman correlation was used. The level of significance is indicated in the figure captions.

## Data Availability

Primary data is available at https://figshare.com/s/4d9bc4da4b7c70e19b8d (DOI: 10.6084/m9.figshare.22561033).

## Declarations

### Inclusion and diversity statement

The inclusion and diversity statement has been filled in.

## Abbreviations

*ACE2*: Angiotensin-converting enzyme 2
*COVID-19*: Coronavirus disease 2019
*CRP*: C-reactive protein
*GFR*: Glomerular filtration rate
*GOT*: Glutamic oxaloacetic transaminase
*IL-6*: Interlekin-6
